# Maize recruits beneficial microorganisms via rhizosphere metabolites as signals to construct a functional network for saline–alkaline stress resistance

**DOI:** 10.3389/fpls.2026.1843423

**Published:** 2026-06-16

**Authors:** Yehui Han, Xinyuan Li, Chao Zhou, Ting Xu, Yue Liu, Yanhui Dou, Guanghui Hu, Junqiang Wang

**Affiliations:** 1 Corn Research Institute, Heilongjiang Academy of Agricultural Sciences, Qiqihar, Heilongjiang, China; 2 College of Life Sciences, Agriculture and Forestry, Qiqihar University, Qiqihar, Heilongjiang, China; 3 Postdoctoral Research Station, Beidahuang Kenfeng Seed Co., Ltd., Harbin, Heilongjiang, China; 4 Corn Research Institute, Heilongjiang Academy of Agricultural Sciences, Harbin, Heilongjiang, China

**Keywords:** maize, metabolomics, microbial co-occurrence network, rhizosphere microbiome, saline–alkaline stress

## Abstract

**Introduction:**

Carbonate-type saline–alkaline stress severely constrains maize production; however, the synergistic response mechanisms between rhizosphere microorganisms and metabolites remain unclear.

**Methods:**

Through field experiments along with the integration of soil chemical factor analysis, microbial high-throughput sequencing, and non-targeted metabolomics, we systematically investigated the response mechanisms of the rhizosphere microecosystem to saline–alkaline stress in maize fields in the carbonate chernozem region of the Songnen Plain, Northeast China.

**Results and discussion:**

Saline–alkaline stress significantly increased soil pH and electrical conductivity (EC) and decreased soil organic matter (SOM), total nitrogen (TN), and total phosphorus (TP) content. However, the rhizosphere exhibited buffering capacity and maintained a high cation exchange capacity (CEC). Microbial community analysis revealed that bacterial alpha diversity increased under stressful conditions. Contrarily, fungal diversity significantly decreased, and the community structure shifted towards a pathogen-dominated community, primarily within Ascomycota, particularly in the genus *Fusarium*. This indicates differential stress tolerance between the bacterial and fungal communities. Co-occurrence network analysis further indicated that saline–alkaline conditions enhanced bacterial network complexity and connectivity, whereas they resulted in the contraction and structural simplification of fungal networks. Metabolite analysis showed that saline–alkaline stress induced significant reprogramming of the rhizosphere metabolic profile. Organophosphorus compounds, nucleotides, and their analogs were significantly enriched, whereas defensive secondary metabolites, such as cajanol, specifically accumulated in the saline–alkaline rhizosphere. Pathway analysis indicated the activation of stress resistance and oxidative stress-mitigation-related pathways, including betalain biosynthesis, flavonoid biosynthesis, tryptophan metabolism, and arginine metabolism. Multi-omics integration analysis identified soil EC and total potassium (TK) as key environmental factors driving the differentiation of microbial and metabolite communities. Key differential metabolites showed significant positive correlations with saline–alkaline-enriched microbial taxa (*Sphingomonas*), revealing a metabolite-mediated microbial recruitment mechanism. Using multi-omics analysis, this study revealed that the maize rhizosphere responds to saline–alkaline stress through metabolic reprogramming (enriching defensive metabolites such as cajanol) to directionally recruit beneficial bacteria such as *Sphingomonas* and maintain a higher bacterial network complexity, while also leading to the pathologization of the fungal community. Our findings highlight that maize recruits beneficial microbes through rhizosphere metabolic reprogramming, providing a mechanistic basis for microbiome-assisted saline–alkaline soil remediation.

## 1 Introduction

Soil salinization–alkalization presents considerable challenges to both global food security and the sustainability of agricultural ecosystems, affecting over 1 billion ha worldwide (Deepthi et al., 2021). Approximately 5% of China’s arable land is affected by saline–alkaline stress (Hou et al., 2022). The Songnen Plain in northeastern China, a major maize (*Zea mays* L.) producing region, is predominantly characterized by widely distributed carbonate-dominated alkaline soils (Wu et al., 2022). Climate change is exacerbating soil degradation and freshwater scarcity (Deepthi et al., 2021; Kumar et al., 2023). Therefore, a comprehensive understanding of the saline–alkaline tolerance mechanisms of crops grown on salinized–alkaline farmland is of strategic importance for achieving national food self-sufficiency goals (Luo et al., 2023).

Carbonate-type alkali stress differs fundamentally from neutral salt stress. The high pH environment, primarily resulting from the prevalence of NaHCO_3_ and Na_2_CO_3_, leads to the fixation of soil nutrients, disrupts cell membrane structures, and induces a series of physiological challenges in plants, including oxidative stress (Gong et al., 2013). Maize is the most important cereal crop in China, accounting for 40% of national production, and exhibiting moderate salt tolerance; however, under severe stress conditions, the yields in carbonate chernozem soils with pH values of 8.2–10.2 can decrease by 30–70% (Wu et al., 2022). Traditional physical-chemical remediation measures, such as leaching and amendment addition, are frequently constrained by cost and long-term effectiveness (Kumar et al., 2023). Consequently, harnessing the rhizosphere microbiome to enhance crop saline–alkaline tolerance has emerged as an important strategy for advancing sustainable agriculture (Wei et al., 2024).

The rhizosphere microbiome facilitates plant adaptation to saline–alkaline stress through various mechanisms, including the synthesis of extracellular polysaccharides to maintain ion homeostasis, secretion of organic acids to mobilize fixed phosphorus, production of phytohormones to alleviate stress, and formation of biofilms to improve soil structure (Yue et al., 2024; Violeta et al., 2022). *Sphingomonas* strains can significantly promote plant growth in saline–alkaline soils (Li et al., 2024). *Bacillus altitudinis* can produce various growth-promoting substances to regulate plant endogenous hormone levels, cell division and differentiation, photosynthesis, and antioxidant capacity, and notably increase plant biomass, metabolites (soluble proteins and sugars), and secondary metabolite content, thereby enhancing the saline–alkaline tolerance of alfalfa (*Medicago sativa* L.) (Violeta et al., 2022). Inoculation with *Funneliformis mosseae* can activate the rhizosphere microbial synergistic network, substantially increasing alfalfa biomass in saline soil (Zhang Z. et al., 2024). Additionally, increasing saline–alkaline concentrations are associated with a decrease in soil microbial community richness and diversity; however, *Sphingomonas* exhibits considerable tolerance under these conditions (Zhang G. et al., 2024).

However, microorganisms are not the only factor influencing plant performance under stress. The rhizosphere metabolome, particularly the assemblage of root exudates and microbial metabolites, constitutes the key chemical basis for plant-microbe interactions. These metabolites (organic acids, amino acids, and compatible solutes) are involved in pH regulation, nutrient activation, signal transduction, and oxidative stress mitigation, serving as key bridges linking microbial functions to plant phenotypes. Shao et al. (2025) demonstrated that planting *Helianthus tuberosus* ameliorated saline–alkaline soils by facilitating the selective enrichment of key microorganisms through key metabolites such as trehalose-6-phosphate (Shao et al., 2025). Li et al. (2023) indicated that *Melia azedarach* demonstrates the capacity to adapt to salt stress when cultivated in saline–alkaline conditions through the regulation of metabolites, such as sugars, amino acids, and flavonoids.

Although previous studies have revealed the partial functions of microorganisms or metabolites, the mechanisms by which microbes and metabolites synergistically facilitate crop adaptation in carbonate-type saline–alkaline soils remain poorly understood at a systematic level. Addressing this knowledge gap requires comprehensive multi-omics analyses that integrate soil chemical properties, microbiomes, and metabolomes under field conditions.

Therefore, this study focused on the carbonate-type saline–alkaline soil in Qiqihar City, Heilongjiang Province. Through field experiments combined with soil chemical factor analysis, microbial high-throughput sequencing, and non-targeted metabolomics, we systematically constructed a soil environment-microbial community-rhizosphere metabolite interaction framework. This study aimed to comprehensively reveal the ecological regulatory network of the maize rhizosphere in response to carbonate-type saline–alkaline stress from an integrated multi-omics perspective, providing new theoretical and empirical foundations for understanding crop-microbe interaction mechanisms and the sustainable utilization of saline–alkaline land.

## 2 Materials and methods

### 2.1 Experimental site and design

Soil sampling was conducted on October 9, 2025, at the experimental base of the Qiqihar Academy of Agricultural Sciences in the Heilongjiang Province (47°35′ N, 124°32′ E). Rhizosphere and bulk soil samples were collected from both saline–alkaline and conventional plots of maize fields and designated as SR (saline–alkaline rhizosphere), SB (saline–alkaline bulk), CR (conventional rhizosphere), and CB (conventional bulk). Each plot measured 5 m in length and 2.6 m in width, with a plant spacing of 22.5 cm. A randomized block design with three replicates was employed, which resulted in six plots. The two experimental fields were adjacent, with identical topography, soil texture, and preceding crop (maize), differing only significantly in salinity-alkalinity levels.

The maize variety used was Tianyu 108. All treatments received the same fertilizer application rate and timing: a slow-release compound fertilizer (N 26%, P_2_O_5_ 10%, K_2_O 12%) was applied once in spring, simultaneous with seeding. The fertilizer application rate was 750 kg ha^-^². The fields were managed in accordance with agronomic standards.

### 2.2 Soil sampling and analysis

Using a sterilized shovel, soil subsamples from six plants per replicate plot were collected in an S-shaped pattern. The rhizosphere soil (approximately 2 mm adhering to the roots) was collected after shaking off the loosely attached soil (Liu et al., 2021). Bulk soil was collected from the same plot, approximately 20 cm from the plant base at the same soil depth. Six plant samples from each plot were randomly divided into two groups (three plants each). Rhizosphere and bulk soils within the same group were homogenized separately to form one composite sample. Each plot produced two composite rhizosphere soil samples and two composite bulk soil samples, resulting in 24 soil samples for the experiment (two stress levels × two soil compartments × three replicate plots × two composite samples per plot). Samples were immediately flash-frozen in liquid nitrogen and stored at −80 °C. Each sample was divided into two portions. One portion was stored at –80 °C for soil microbial and metabolite extraction, and the remaining soil was air-dried to determine soil chemical properties.

Soil pH was measured using a pH meter (Thermo Orion-868) at a soil-to-water ratio of 1:2.5. The electrical conductivity (EC) was determined at a soil: water ratio of 1:5. The extract was centrifuged, and the EC of the supernatant was measured using a conductivity meter. Cation exchange capacity (CEC) was determined using the ammonium acetate exchange method (pH 7.0). Soil organic matter (SOM) was measured using the potassium dichromate-concentrated sulfuric acid oxidation external heating method. Total nitrogen (TN) was determined using the semi-micro Kjeldahl method (Tan and Troth, 1981). Total phosphorus (TP) was determined colorimetrically with molybdenum blue after extraction with 0.5 mol/L sodium bicarbonate. Total potassium (TK) was extracted using 1 mol/L ammonium acetate and measured using a flame photometer (6400A; INESA, China) (Tao et al., 2025).

### 2.3 Soil DNA extraction and microbial high-throughput sequencing

Total DNA extraction and sequencing of the microbial samples were performed by Majorbio Bio-Pharm Technology Co., Ltd. (Shanghai, China). DNA was extracted from 0.5 g of fresh soil using the E.Z.N.A.^®^ Soil DNA Kit (Omega Bio-tek, Norcross, GA, USA) following the manufacturer’s instructions. The V3–V4 region of the bacterial 16S rDNA was amplified using primers 338F (5′-ACTCCTACGGGAGGCAGCAG-3′) and 806R (5′-GGACTACHVGGGTWTCTAAT-3′) (Huang et al., 2022). The fungal ITS1 region was amplified using primers ITS1F (5′-CTTGGTCATTTAGAGGAAGTAA-3′) and ITS2R (5′-GCTGCGTTCTTCATCGATGC-3′) (Hu et al., 2022). The amplification products were checked for concentration using a NanoDrop 2000 UV-Vis spectrophotometer (Thermo Scientific, Wilmington, DE, USA), verified for quality by 1% agarose gel electrophoresis, and quantified using a QuantiFluor-ST fluorometer (Promega, Madison, WI, USA). Purified amplicons were pooled in equimolar quantities and paired-end sequenced (2 × 300 bp) using the Illumina MiSeq PE300 platform.

Following sequencing, raw FASTQ data were analyzed using the QIIME2 pipeline (version 2022.2). Raw sequence data were demultiplexed using the demux plugin, and primers were trimmed using the Cutadapt plugin (Marcel, 2011). Sequences were subsequently quality-filtered, denoised, and merged, and chimeras were removed using the DADA2 plugin (Callahan et al., 2016). Non-singleton amplicon sequence variants (ASVs) were clustered for a more precise species resolution by accurately distinguishing single-nucleotide differences. Sequences were aligned using Mafft (Katoh et al., 2002). Raw sequences containing ambiguous nucleotides with low-quality scores (Q < 20) or shorter lengths (<50 bp) were discarded. Sequences with an overlap length >16 bp were merged based on their overlap degree, with no more than 5 bp mismatches allowed. Taxonomy was assigned to 16S and ITS sequences (97% similarity) using the classify-sklearn (naive Bayes) method with SILVA (v138) and UNITE (v8.0) databases, respectively. Sequencing libraries were rarefied to the smallest library size among sample groups. Raw sequencing data were deposited in the NCBI Sequence Read Archive (SRA) under the accession number PRJNA1433267.

### 2.4 Metabolite extraction and analysis

An accurately measured 100 mg sample of rhizosphere soil was placed into a centrifuge tube (2 mL), followed by the addition of 1000 μL extract [methanol/water (v/v, 4:1)] containing 20 μg mL^-1^ 2-chloro-L-phenylalanine. Rhizosphere samples were ground for 6 min (50 Hz, –10 °C) using a grinder, followed by ultrasonic extraction for 30 min (40 KHz, 5 °C). Rhizosphere soils were centrifuged at 13,000 rpm for 15 min at 4 °C, and the supernatants were transferred to an injection bottle. The LC-MS/MS analysis of the sample was conducted on a UHPLC-Q Exactive HF-X system equipped with an ACQUITY HSS T3 column (100 × 2.1 mm i.d., 1.8 μm; Waters, USA) at Majorbio Bio-Pharm Technology Co. Ltd. (Shanghai, China). The mobile phases consisted of 0.1% formic acid in water: acetonitrile (2:98, v/v) (solvent A) and 0.1% formic acid in acetonitrile (solvent B). The flow rate was 0.40 mL min^-1^, and the column temperature was 40 °C. The injection volume was 5 μL.

To ensure the stability and accuracy of this study, the extracts of all the rhizosphere samples were mixed in the same volume to prepare quality control samples. The volume, processing, and testing of each quality control sample were the same as those of the analytical rhizosphere samples. In the instrumental analysis, one quality control sample was inserted into every 12 analytical rhizosphere samples to investigate the stability of the entire process.

Pretreatment of raw LC/MS data was performed using Progenesis QI software (Waters Corporation, Milford, USA), and a three-dimensional data matrix in CSV format was exported. The information in this three-dimensional matrix included sample information, metabolite name, and mass spectral response intensity. Internal standard peaks and any known false-positive peaks, including noise, column bleed, and derivatized reagent peaks, were removed from the data matrix, deduplicated, and pooled. Simultaneously, the metabolites were identified by searching the database. The main databases were the Human Metabolome Database (HMDB) (http://www.hmdb.ca/), Metabolite Link (Metlin) (https://metlin.scripps.edu/), and the self-compiled Majorbio Database (MJDB) of Majorbio Biotechnology Co., Ltd. (Shanghai, China).

The data matrix obtained by searching the database was uploaded to the Majorbio Cloud platform (https://cloud.majorbio.com) for data analysis. First, the data matrix was preprocessed by retaining only those metabolic features that were detected in at least 80% of any set of samples. After filtering, the minimum value in the data matrix was selected to impute missing values, and each metabolic signature was normalized to the sum. To reduce errors caused by sample preparation and instrument instability, the response intensities of the sample mass spectrometry peaks were normalized using the sum normalization method to obtain a normalized data matrix. Variables of quality control (QC) samples with a relative standard deviation (RSD) of > 30% were excluded and log10 logarithmicized to obtain the final data matrix for subsequent analysis. A variance analysis of the matrix file was performed after data preprocessing. The metabolomics data have been deposited in the MetaboLights repository with the study identifier MTBLS13956.

### 2.5 Co-occurrence network analysis

Microbial co-occurrence networks were calculated and initially plotted using the igraph package in R (version 4.3.1), and further visualized using Gephi. Bacterial and fungal co-occurrence networks at the ASV level were assessed using Spearman’s rank correlation (Shi et al., 2023). To construct robust co-occurrence networks, we first filtered out low-abundance or low-prevalence ASV, retaining only those with a relative abundance of >0.1% for analysis. Networks were constructed based on Spearman correlations (|r| > 0.7, *P* < 0.05), and p-values were subsequently adjusted using false discovery rate (FDR) correction to control for false-positive associations. Network complexity was evaluated using the node number, edge number, average clustering coefficient, average degree, and graph density. Positive and negative correlations between nodes represent cooperative and competitive interactions, respectively (Wang et al., 2024a).

### 2.6 Statistical analysis

Based on representative sequences and abundance information, a series of statistical analyses and visualizations were performed using R (version 4.3.1). Prior to the analysis, the Variance Inflation Factor (VIF) analysis was conducted to ensure that multicollinearity did not affect the final results. Alpha diversity indices (Sobs and Shannon) for the bacterial and fungal communities were calculated using the vegan package (Oksanen et al., 2018). Principal coordinate analysis (PCoA) and canonical correspondence analysis (CCA) based on Bray–Curtis distance matrices were performed to determine the effects of saline–alkaline stress on bacterial and fungal community composition (Gower, 1966). The relative abundance distribution patterns of bacteria and fungi were visualized using the ggplot2 package (Ginestet, 2011). One-way ANOVA with Tukey’s honest significant difference (HSD) test in the agricolae package was used to assess differences in soil chemical properties and diversity indices. Principal component analysis (PCA) and orthogonal partial least squares discriminant analysis (OPLS-DA) were performed using the ropls package (version 1.6.2) for metabolomics data. Model stability was evaluated via seven-fold cross-validation. Differential metabolites were screened based on a variable importance in projection (VIP) score > 1 from the OPLS-DA model and a p-value < 0.05 using Student’s t-test. To reveal the intrinsic links among the multi-omics data, Spearman’s correlation analysis was performed using the pheatmap package (v1.0.8). Procrustes analysis in the vegan package was used to rotate and superimpose one PCA ordination plot onto another, visualize the overall similarity between different datasets, and elucidate significant correlations among soil chemical properties, differential microbial taxa, and key metabolites (Strom et al., 2020).

## 3 Results

### 3.1 Divergence in soil physicochemical properties under saline–alkali stress

The physicochemical profile of the experimental site revealed a distinct environmental gradient driven by carbonate-type salinity (**Table 1**). As expected, the saline–alkali soils (SR and SB) exhibited significantly higher pH values (8.48–8.66) and EC (163.00–185.83 mS m^-1^) than those of the control plots (CR and CB), indicating the severity of the stress conditions.

**Table 1:** Differential analysis of soil chemical properties under different treatments.

Treatment	pH	EC (mS m^-1^)	CEC (cmol^+^ kg^-1^)	SOM (g kg^-1^)	TP (%)	TK (%)	TN (mg kg^-1^)
CR	8.36 ± 0.012d	97.27 ± 0.19c	27.20 ± 0.33a	31.23 ± 1.04c	0.084 ± 0.00075a	2.17 ± 0.018c	455 ± 3.1c
CB	8.58 ± 0.010b	97.75 ± 0.16c	7.37 ± 0.26b	49.70 ± 1.49a	0.082 ± 0.0014b	2.14 ± 0.012d	762 ± 4.6a
SR	8.48 ± 0.014c	163.00 ± 1.14b	27.23 ± 0.12a	25.47 ± 0.99d	0.057 ± 0.0012c	2.44 ± 0.010b	480 ± 2.1b
SB	8.66 ± 0.014a	185.83 ± 1.47a	6.62 ± 0.19c	39.17 ± 1.53b	0.055 ± 0.0012d	2.47 ± 0.012a	432 ± 2.6d

CR/CB, Conventional plot rhizosphere/bulk soil; SR/SB, Saline-alkali plot rhizosphere/bulk soil. Values are mean ± SD (n=6). Different lowercase letters within a column indicate significant differences at *P* < 0.05 based on Tukey’s HSD test.

Salinization profoundly altered nutrient stoichiometry. TN, TP, and SOM were notably depleted in the saline soils. The SOM content in SB was 39.17 g kg^-1^, which was significantly lower than the 49.70 g kg^-1^ observed in CB. Notably, the rhizosphere effect partially mitigated these deficits; the CEC in the SR was maintained at levels comparable to those in the CR, indicating that maize root activity may actively recruit ions or secrete exudates to buffer the immediate root environment against ion toxicity. However, although CEC was maintained in the rhizosphere samples, SOM still declined under stress conditions, indicating that the rhizosphere buffering effect is limited to a certain extent.

### 3.2 Bacterial community diversity, structure, and taxonomic composition

**Figure 1:**
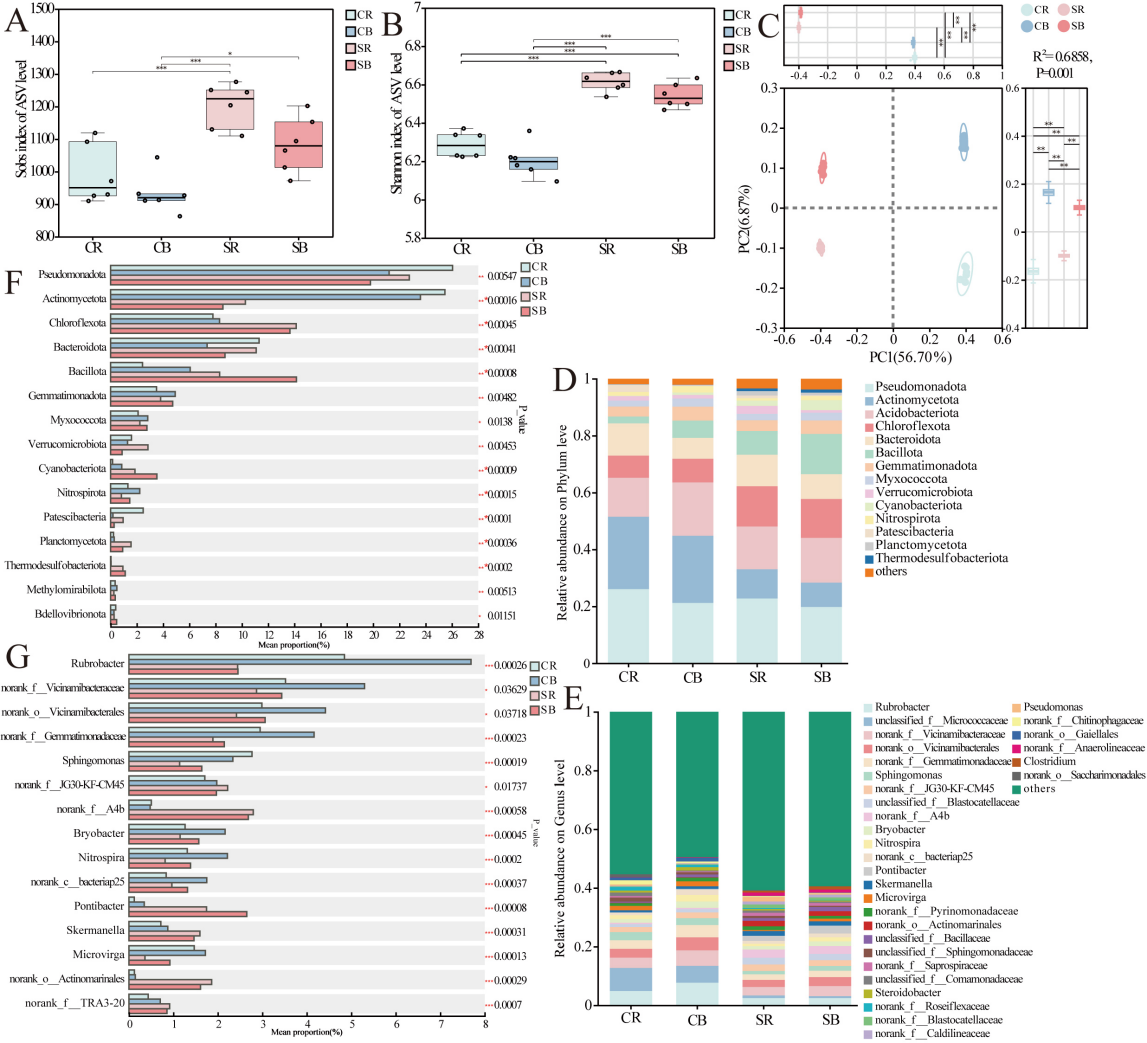
Characteristics of bacterial community diversity, structure, and taxonomic composition. **(A, B)** Bacterial alpha diversity indices (Shannon and Sobs); **(C)** PCoA analysis of bacterial communities based on Bray–Curtis distance; **(D, F)** Community composition at the phylum level and significance testing; **(E, G)** Community composition at the genus level and comparison of significantly differential genera. CR/CB, Conventional plot rhizosphere/bulk soil; SR/SB, Saline-alkali plot rhizosphere/bulk soil. *P < 0.05, **P < 0.01, ***P < 0.001, Kruskal-Wallis H test.

High-throughput sequencing revealed significant responses of bacterial community diversity and structure to saline–alkaline stress and the rhizosphere effect. Contrary to soil nutrient trends, the alpha diversity indices of bacterial communities were significantly higher in saline–alkali soils than in the control soils (**Figures 1A, B**). Both Sobs and Shannon indices for the SR and SB treatments were significantly higher than those for the CR and CB treatments. Specifically, the SR treatment exhibited the highest Sobs and Shannon indices, indicating that the saline–alkaline rhizosphere environment recruited a more diverse bacterial community.

PCoA at the ASV level (**Figure 1C**) revealed an apparent separation pattern in microbial communities. The first principal component (PC1, explaining 56.70% of the variation) primarily separated samples based on soil salinity status (control vs. saline–alkali), whereas the second component (PC2, explaining 6.87% of the variation) distinguished rhizosphere from bulk soil niches. PERMANOVA further revealed highly significant differences in community structure among treatments (*R²* = 0.6858, *P* = 0.001). The marginal boxplots in **Figure 1C** statistically validate this separation, showing significant differences (*P* < 0.01) between the saline–alkali (SR, SB) and control groups (CR, CB) along the PC1 axis, and between the rhizosphere and bulk soil within the control group along the PC2 axis.

Taxonomic classification at the phylum level (**Figure 1D**) revealed that the bacterial community was primarily dominated by Pseudomonadota, Acidobacteriota, Actinomycetota, Chloroflexota, and Bacteroidota. Differential statistical analysis (**Figure 1F**) indicated that saline–alkaline stress significantly altered the community composition at the phylum level. Compared with the control, the relative abundance of Pseudomonadota significantly increased in saline–alkali soils (SR and SB). Similarly, Chloroflexota, Bacteroidota, and Gemmatimonadota were significantly enriched under the saline–alkali treatment. Conversely, Actinomycetota and Acidobacteriota were significantly reduced in the SR and SB, indicating a preference for non-saline–alkaline environments. Notably, Bacillota exhibited a unique enrichment in the SB treatment.

At the genus level (**Figure 1E**), community composition showed apparent heterogeneity among the treatments. Statistical comparison of the dominant genera (**Figure 1G**) further identified the specific taxa responsible for the observed compositional heterogeneity among treatments. The dominant genus *Rubrobacter* in the control soil was significantly suppressed by SR and SB treatments. In contrast, taxa such as *norank_f:Vicinamibacteraceae* and *Pontibacter* were significantly enriched in the saline–alkali group. The rhizosphere effect under saline–alkaline conditions was particularly evident for *Sphingomonas*, which showed the highest relative abundance in SR, followed by SB, and was the lowest in the controls. Additionally, *Bryobacter* and *Nitrospira* were more abundant in the control soils, whereas *Microvirga* and *Skermanella* were more abundant in the saline–alkali soils.

### 3.3 Fungal community diversity, structure, and taxonomic composition

**Figure 2:**
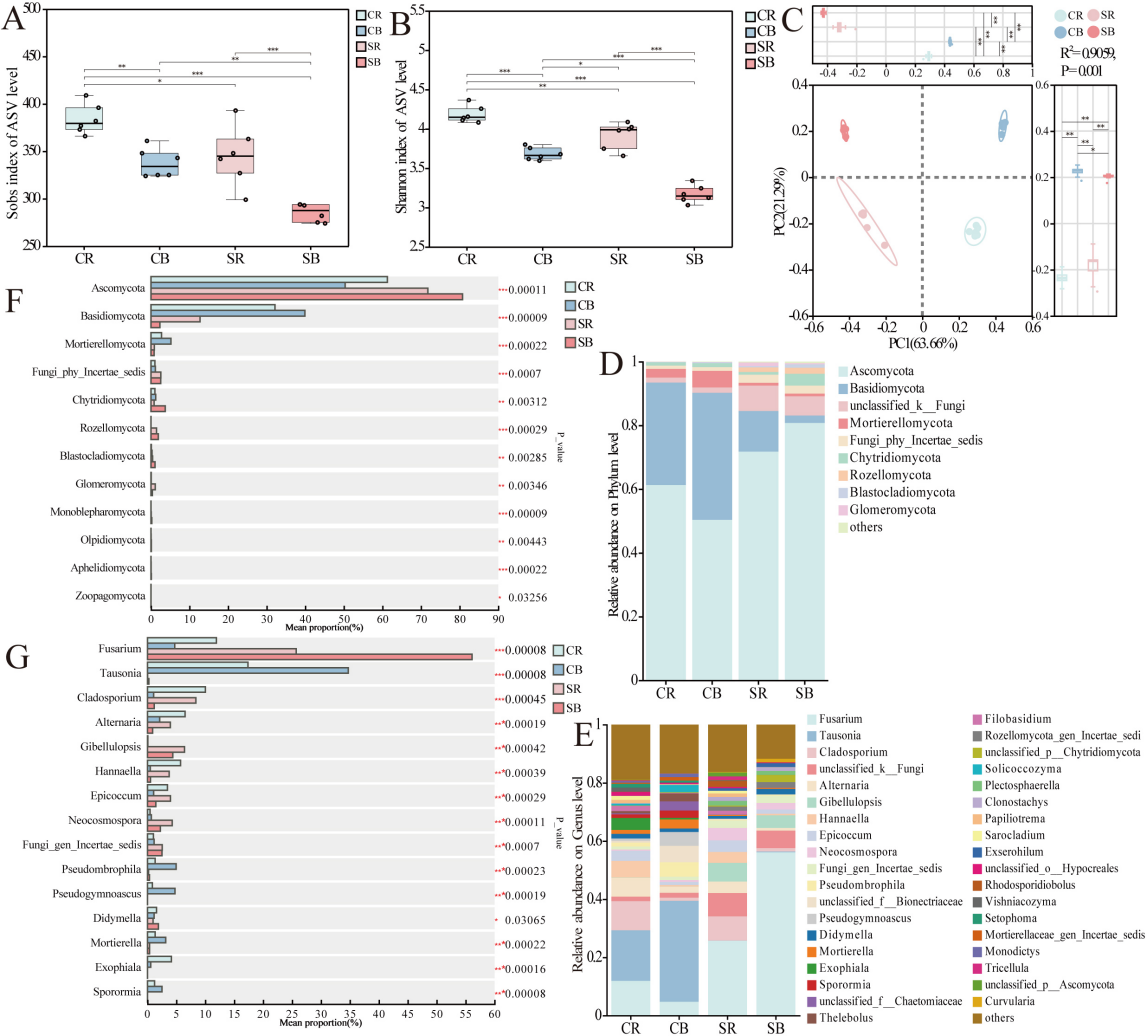
Characteristics of fungal community diversity, structure, and taxonomic composition. **(A, B)** Fungal alpha diversity indices (Shannon and Sobs); **(C)** PCoA analysis of fungal communities based on Bray–Curtis distance; **(D, F)** Community composition at the phylum level and significance testing; **(E, G)** Community composition at the genus level and comparison of significantly differential genera. CR/CB, Conventional plot rhizosphere/bulk soil; SR/SB, Saline-alkali plot rhizosphere/bulk soil. * *P* < 0.05, ** *P* < 0.01, *** *P* < 0.001, Kruskal-Wallis H test.

In contrast to the bacterial response, the fungal community exhibited a significant decrease in diversity and a notable structural shift under saline–alkaline stress (**Figures 2A, B**). The Sobs index was the highest in the CR group and the lowest in the SB group. The Sobs index for SB was significantly lower than those for CR and SR. Similarly, the Shannon index indicated that saline–alkaline stress significantly inhibited fungal diversity. The CR treatment maintained the highest diversity level, which was significantly higher than those of the CB, SR, and SB treatments. Notably, under saline–alkaline conditions, the rhizosphere effect (SR) partially alleviated diversity loss, and its diversity was significantly higher than that of bulk soil (SB).

PCoA at the ASV level revealed an apparent separation of the fungal communities influenced by soil conditions (**Figure 2C**). The first two principal coordinates explained 63.66% and 21.29% of the variation. The fungal community structure was more strongly driven by environmental gradients than the bacterial community, as reflected by the higher explained variance. Samples were clustered into two distinct groups along the PC1 axis, completely separating the saline–alkali soils (SR and SB) from the control soils (CR and CB). Marginal boxplots showed significant differences (*P* < 0.01) along both the PC1 (salinity effect) and PC2 (rhizosphere effect) axes. PERMANOVA further validated the highly significant differences in the fungal community structure among treatments, with a very high effect size (*R²* = 0.9059, *P* = 0.001).

Taxonomic classification at the phylum level (**Figure 2D**) showed that Ascomycota and Basidiomycota were the dominant phyla in all samples. However, their relative proportions changed considerably under stress conditions. Differential statistical analysis (**Figure 2F**) showed that Ascomycota was significantly enriched and emerged as the dominant group in the saline–alkali treatments (SR and SB) compared with the control. Conversely, the abundance of Basidiomycota was significantly lower in the saline–alkali soils than in the control soils. Additionally, minor phyla such as Chytridiomycota and Glomeromycota were significantly more abundant in the control treatment.

At the genus level (**Figure 2E**), community composition showed strong niche-specific patterns. CR and CB were characterized by diverse genera such as *Tausonia*, *Cladosporium*, and *Alternaria*. In contrast, saline–alkali soils were mainly dominated by Fusarium. Statistical comparison of the dominant genera (**Figure 2G**) quantified these differences, identifying Fusarium as the most significant biomarker in the saline–alkaline environment, with its relative abundance in SR and SB being significantly higher than in CR and CB. In contrast, the genera *Tausonia*, *Cladosporium*, and *Alternaria* were significantly enriched in the control group. *Mortierella* also showed a significant preference for the control environment.

### 3.4 Identification of differential microbial taxa and biomarkers

Linear discriminant analysis effect size (LEfSe) was performed (LDA threshold > 4.0) to identify specific bacterial and fungal taxa that significantly distinguished microbial communities across different soil niches.

**Figure 3:**
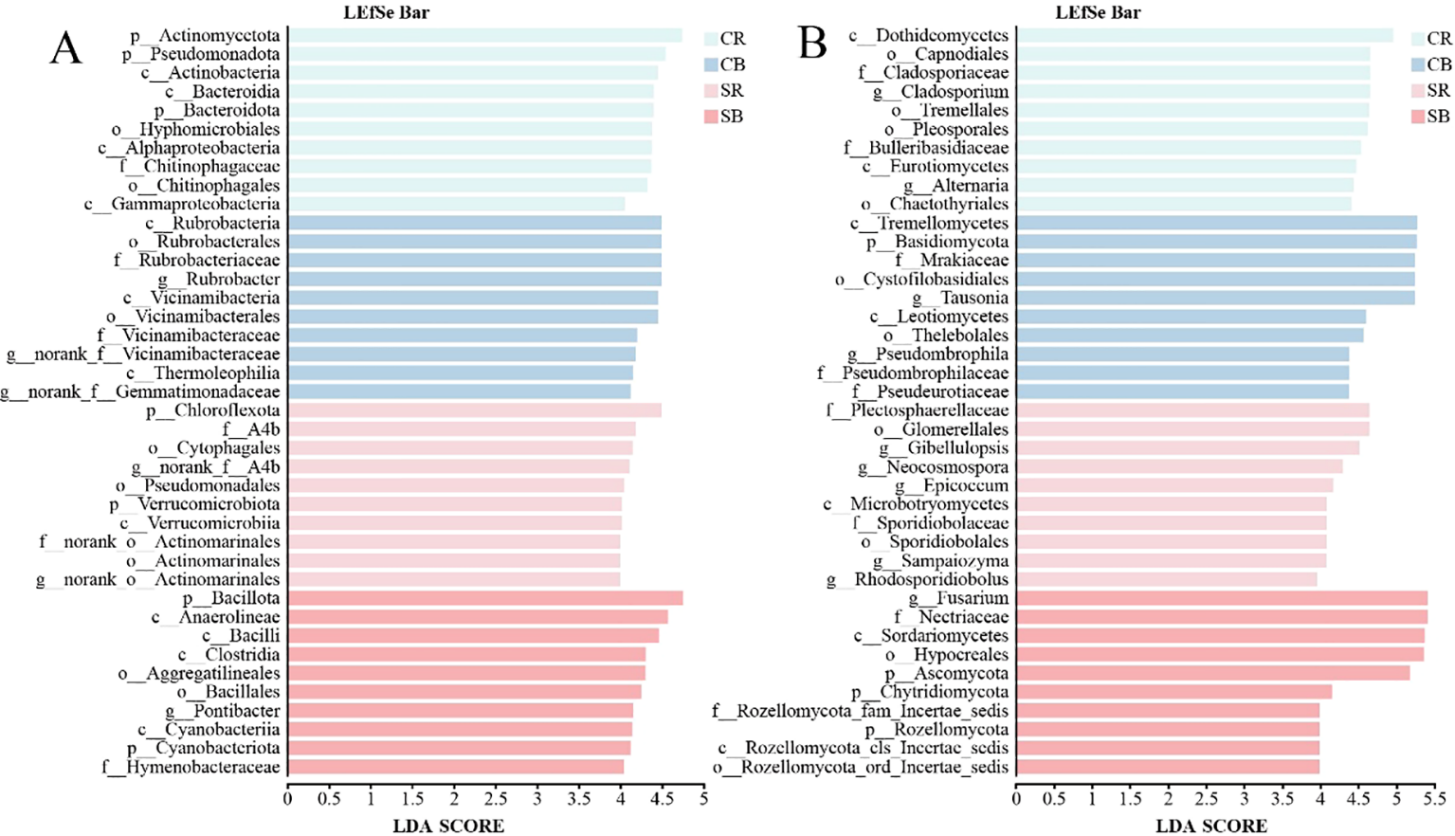
LEfSe identification of biomarkers for bacterial **(A)** and fungal **(B)** communities. Taxa with significantly different LDA scores>4.0 in different treatments are shown. CR/CB, Conventional plot rhizosphere/bulk soil; SR/SB, Saline-alkali plot rhizosphere/bulk soil.

Unique biomarkers of the bacterial community (**Figure 3A**) were identified in each treatment group. The CR was primarily enriched in Actinomycetota, Pseudomonadota, Alphaproteobacteria, and Hyphomicrobiales. In CB, Rubrobacteria and its corresponding genus *Rubrobacter* were the most significant biomarkers, with high LDA scores (> 4.5). The SR was characterized by the enrichment of Chloroflexota and Cytophagales. In contrast, SB exhibited a unique taxonomic signature dominated by Bacillota and Bacilli, with the highest LDA scores among all the groups. Additionally, *Pontibacter*, Bacillales, and Cyanobacteria were significantly enriched in SB.

For the fungal community (**Figure 3B**), the separation of differential taxa was more pronounced, particularly at the phylum and genus levels. CR was significantly enriched in Capnodiales, *Cladosporium*, and *Alternaria*. CB was significantly enriched in Basidiomycota and Tremellomycetes, with *Tausonia* as the primary biomarker (LDA > 4.5). Under saline–alkaline conditions, the fungal markers underwent a substantial shift. The SR was enriched in *Gibellulopsis*, *Neocosmospora*, and *Epicoccum*. SB was dominated by Ascomycota and *Fusarium*. The LDA score for Fusarium exceeded 5.0, indicating that it was the strongest biomarker distinguishing the saline–alkaline bulk soil environment from the others. Other stress-related taxa, such as Nectriaceae and Sordariomycetes, were also significantly enriched in the SB group.

### 3.5 Functional prediction of microbial communities

The bacterial metabolic pathways and fungal trophic modes were predicted to provide insights into the functional potential of the rhizosphere microbiome under saline–alkaline stress.

**Figure 4:**
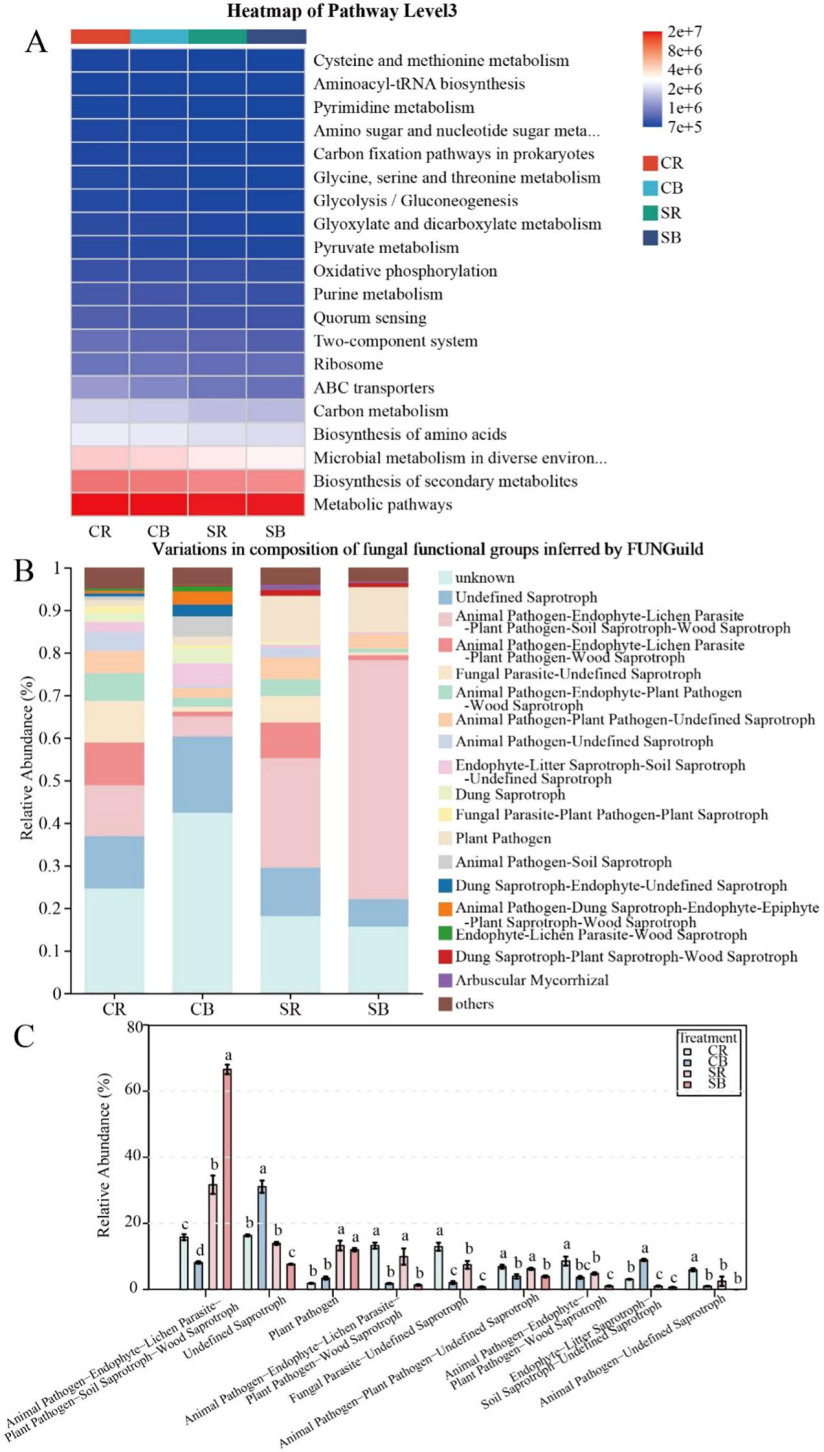
Functional prediction of microbial communities. **(A)** Heatmap of bacterial KEGG Level 3 metabolic pathways. **(B)** Prediction of fungal trophic modes based on FUNGuild. **(C)** Significant differences in fungal trophic modes among different treatments. CR/CB, Conventional plot rhizosphere/bulk soil; SR/SB, Saline-alkali plot rhizosphere/bulk soil. Different lowercase letters within a column indicate significant differences at *P* < 0.05 based on Tukey’s HSD test.

The Kyoto Encyclopedia of Genes and Genomes (KEGG) pathways at Level 3 heatmap indicated that although the overall metabolic functional profile remained conserved, specific stress-responsive shifts were observed among the treatments (**Figure 4A**). Across all samples, “Metabolic pathways,” “Biosynthesis of secondary metabolites,” and “Microbial metabolism in diverse environments” were the most abundant functional categories. However, apparent variation patterns emerged in response to the soil conditions. Pathways related to environmental information processing and membrane transport, particularly “ABC transporters” and “Two-component system,” exhibited higher relative abundances in saline–alkali treatment groups compared to the lower levels in controls. Conversely, pathways associated with rapid growth and biosynthesis, such as “Ribosome” and “Aminoacyl-tRNA biosynthesis,” had relatively lower abundances in saline–alkali samples.

Fungal Functional Guild (FUNGuild) analysis revealed a profound reorganization of fungal trophic modes driven by both salinity and rhizosphere effects (**Figures 4B, C**). In the control treatments, the fungal communities were predominantly composed of saprotrophs. In the bulk soil of the CB, “Undefined Saprotroph” was the main dominant functional guild, occupying the largest proportion of the community, followed by “Dung Saprotroph–Undefined Saprotroph.” The CR maintained a high proportion of saprotrophs; however, compared with its corresponding bulk soil, a significant increase in the proportion of “Animal Pathogen–Undefined Saprotroph” was observed. In contrast, saline–alkali stress induced a notable shift toward pathotrophic and polyphagous trophic guilds. In the SB, the relative abundance of “Undefined Saprotroph” decreased to its lowest level. Rather, the community was mainly dominated by the complex multifunctional guild “Animal Pathogen–Endophyte–Lichen Parasite–Plant Pathogen–Wood Saprotroph,” which accounted for more than 60% of the total fungal community. The SR exhibited a transitional pattern: although it retained a portion of saprotrophic functions, there was significant enrichment of guilds related to “Plant Pathogen” and “Fungal Parasite” compared to that of the control rhizosphere.

### 3.6 Characteristics of microbial co-occurrence networks

**Figure 5:**
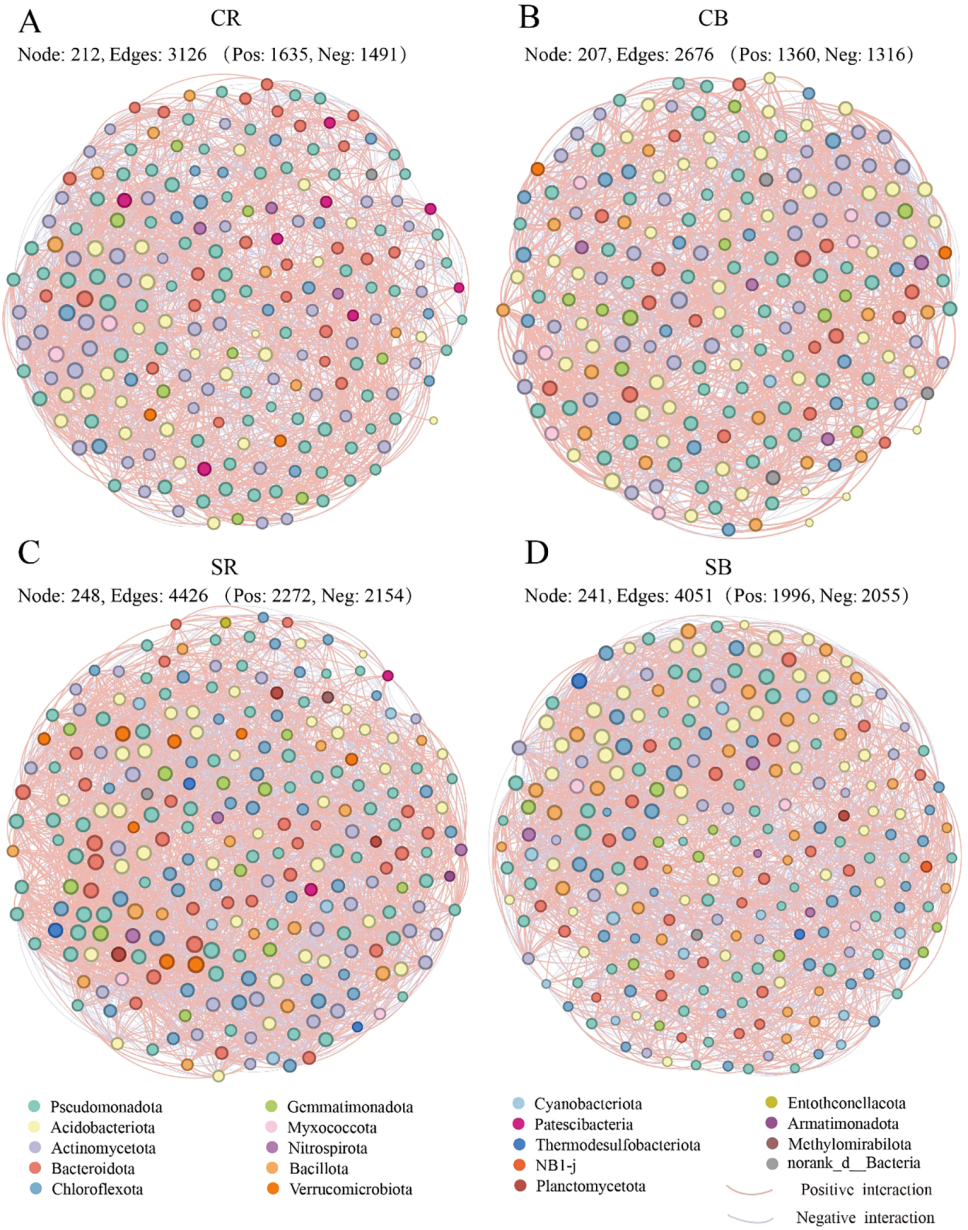
Visualization of bacterial community co-occurrence networks under different treatments **(A–D)**. Nodes represent different species (ASVs), colors correspond to phylum-level classification, with size indicating the relative abundance of the corresponding ASV; edges represent significant correlations between species. CR/CB, Conventional plot rhizosphere/bulk soil; SR/SB, Saline-alkali plot rhizosphere/bulk soil.

**Figure 6:**
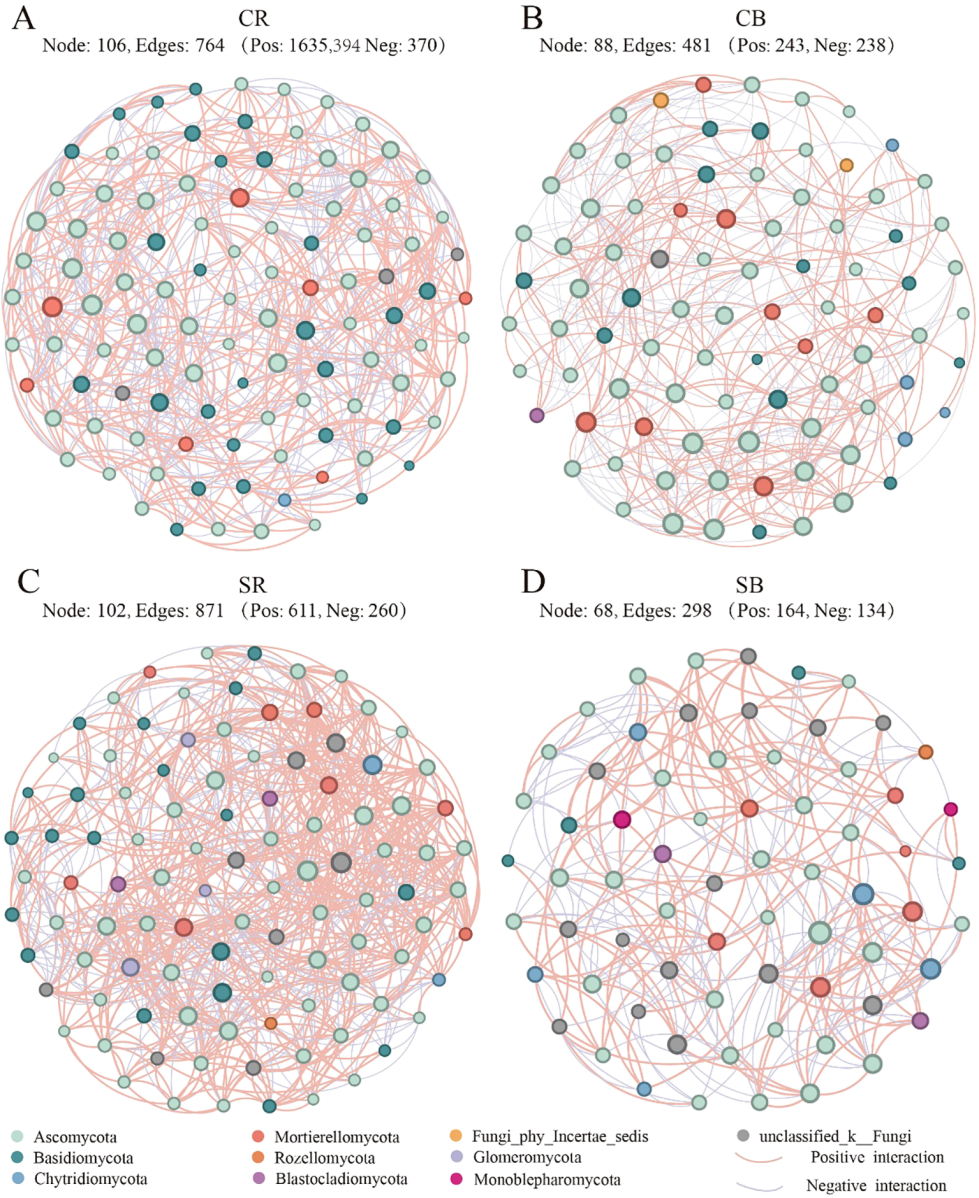
Visualization of fungal community co-occurrence networks under different treatments **(A–D)**. Nodes represent different species (ASVs), colors correspond to phylum-level classification, with size indicating the relative abundance of the corresponding ASV; edges represent significant correlations between species. CR/CB, Conventional plot rhizosphere/bulk soil; SR/SB, Saline-alkali plot rhizosphere/bulk soil.

To evaluate the influence of saline–alkaline stress and rhizosphere effects on microbial community interactions, co-occurrence networks for bacteria and fungi were constructed, and their topological properties were analyzed (**Table 2**; **Figures 5**, **6**).

**Table 2:** Main topological parameters of bacterial and fungal co-occurrence networks.

Microbial species	Treatment	Average degree	Network density	Average clustering coefficient
Bacteria	CR	29.491	0.14	0.513
	CB	25.855	0.126	0.491
	SR	35.694	0.145	0.511
	SB	33.618	0.14	0.515
Fungi	CR	14.415	0.137	0.503
	CB	10.932	0.126	0.504
	SR	17.078	0.169	0.556
	SB	8.765	0.131	0.484

CR/CB, Conventional plot rhizosphere/bulk soil; SR/SB, Saline-alkali plot rhizosphere/bulk soil.

Saline–alkaline stress significantly enhanced the complexity of bacterial networks (**Figure 5**). Compared with the control groups (CR and CB), the bacterial networks in the saline–alkali treatment groups (SR and SB) had a higher average degree, network density, and average clustering coefficient. Specifically, the average degree increased from 29.491 in CR to 35.694 in SR, indicating enhanced bacterial network complexity under saline-alkaline stress. Specifically, the saline–alkali rhizosphere soil (SR) exhibited the most complex network structure, with the highest average degree (35.694) and a significantly higher number of edges (4426) than those of the other treatments.

For fungal networks, the response to saline–alkaline stress differed from that of bacteria and showed evident niche differentiation (**Figure 6**). The rhizosphere effect significantly increased fungal network connectivity, with the SR treatment achieving the highest average degree (17.078) and number of edges (871). However, the fungal network in SB was significantly contracted, with the lowest number of nodes (68), edges (298), and average degree (8.765) among all treatments.

Thus, saline-alkaline conditions enhanced the complexity and connectivity of bacterial networks, whereas they resulted in the contraction and structural simplification of fungal networks, and the rhizosphere effect was relatively evident.

### 3.7 Rhizosphere metabolome reprogramming and key metabolic drivers

This study employed non-targeted metabolomics (LC-MS) to analyze the chemical interactions between maize roots and the soil microbiome under saline–alkaline stress.

**Figure 7:**
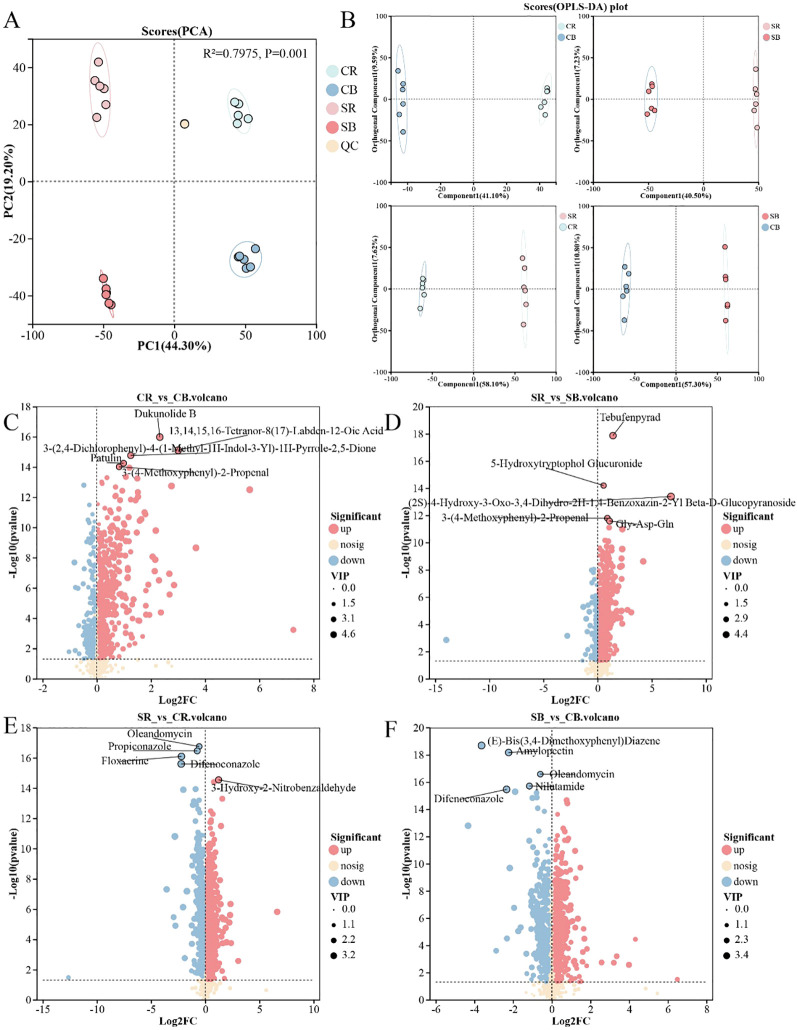
Multivariate statistical analysis and differential metabolite screening of the rhizosphere metabolome. **(A)** PCA score plot of metabolic profiles; **(B)** OPLS-DA model validation plot; **(C–F)** Volcano plots of differential metabolites for different pairwise comparison groups, with red representing upregulation and blue representing downregulation. CR/CB, Conventional plot rhizosphere/bulk soil; SR/SB, Saline-alkali plot rhizosphere/bulk soil.

PCA revealed an apparent separation of metabolic profiles driven by soil salinity and niche differentiation (**Figure 7A**). The first two principal components explained 63.50% of the total variation. PC1 (44.30%) distinctly separated SR and SB from CR and CB, whereas PC2 (19.20%) differentiated SR and CR from SB and CB. Tight clustering of QC samples indicated the stability and reproducibility of the instrumental analysis. To further maximize the intergroup discrimination, orthogonal partial least squares-discriminant analysis (OPLS-DA) was performed (**Figure 7B**). An evident separation was observed in all pairwise comparison groups (CR vs. CB, SR vs. SB, SR vs. CR, and SB vs. CB), with a high explained variance for the first predictive component (ranging from 40.50% to 58.10%), indicating that both salt stress and root exudation induced substantial metabolic reprogramming.

Univariate analysis (volcano plots) visually showed significant differential metabolites (VIP > 1, *P* < 0.05) between the treatments. In the control treatment (CR vs. CB; **Figure 7C**), Dukunolide B and patulin were significantly enriched in the rhizosphere compared to the corresponding bulk soil. Under saline–alkaline conditions (SR vs. SB, **Figure 7D**), tebufenpyrad, 5-hydroxytryptophol glucuronide, and Gly-Asp-Gln were specifically enriched in the rhizosphere. Notably, a comparison between the saline and alkaline and control rhizospheres (SR vs. CR, **Figure 7E**) revealed the specific effect of salt stress on root exudation. Compounds such as 3-hydroxy-2-nitrobenzaldehyde were significantly enriched in the saline–alkaline rhizosphere. Conversely, oleandomycin, propiconazole, and floxacrine were significantly less abundant in the saline-alkaline rhizosphere than in the control rhizosphere. In the bulk soil comparison (SB vs. CB, **Figure 7F**), (E)-bis(3,4-dimethoxyphenyl) diazene and amylopectin were the most significantly less abundant metabolites in the saline–alkali group.

**Figure 8:**
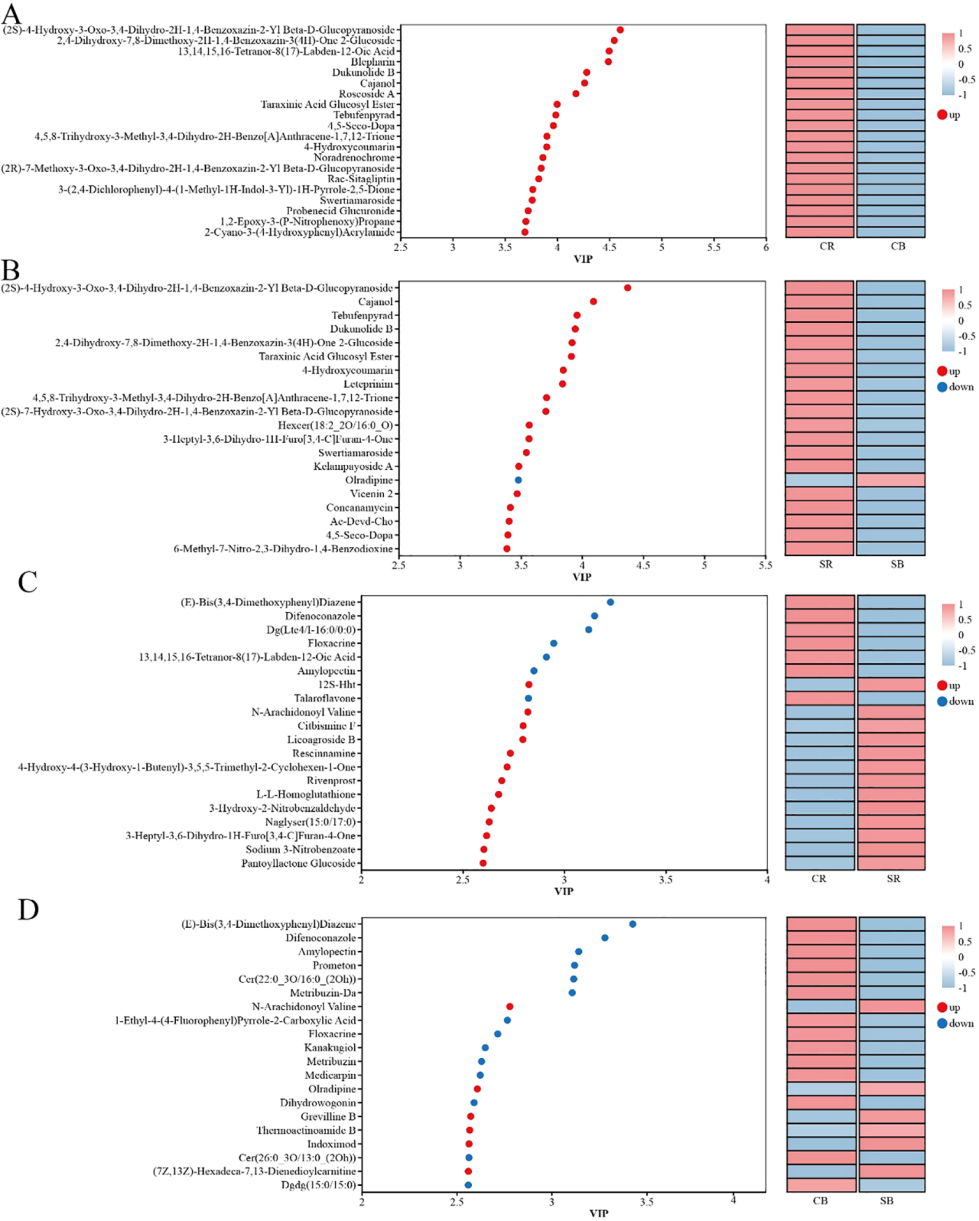
VIP score distribution of key differential metabolites between treatments. **(A–D)** List the top 20 key metabolites with VIP > 2.5 in CR vs CB, SR vs SB, SR vs CR, and SB vs CB comparisons, respectively. The heatmap on the right shows the relative abundance changes of these metabolites in the corresponding treatment groups. CR/CB, Conventional plot rhizosphere/bulk soil; SR/SB, Saline-alkali plot rhizosphere/bulk soil.

VIP score plots identified the top differential metabolites that contributed to group separation. In the CR vs. CB comparison (**Figure 8A**), blepharin, Dukunolide B, cajanol, Roseoside A, and taraxinic acid glucosyl ester were the metabolites with the highest VIP scores, and were all significantly upregulated. Among these, blepharin belongs to glycoside compounds, cajanol is an isoflavonoid compound, and roseoside A is a sesquiterpenoid compound. In the SR vs. SB comparison (**Figure 8B**), (2S)-4-hydroxy-3-oxo-3,4-dihydro-2H-1,4-benzoxazin-2-yl β-D-glucopyranoside and cajanol were identified as key biomarkers with VIP scores > 4.0, both of which were highly enriched in the SR group. In the SR vs. CR comparison (**Figure 8C**), the metabolic shift was characterized by the downregulation of difenoconazole, floxacrine, and amylopectin in the saline–alkaline rhizosphere. These metabolites belong to triazole, dihydroacridinedione, and polysaccharide compounds, respectively. Similarly, difenconazole, amylopectin, and prometon were the primary contributors distinguishing SB from CB (**Figure 8D**), showing reduced abundance in the saline–alkaline bulk soil.

**Figure 9:**
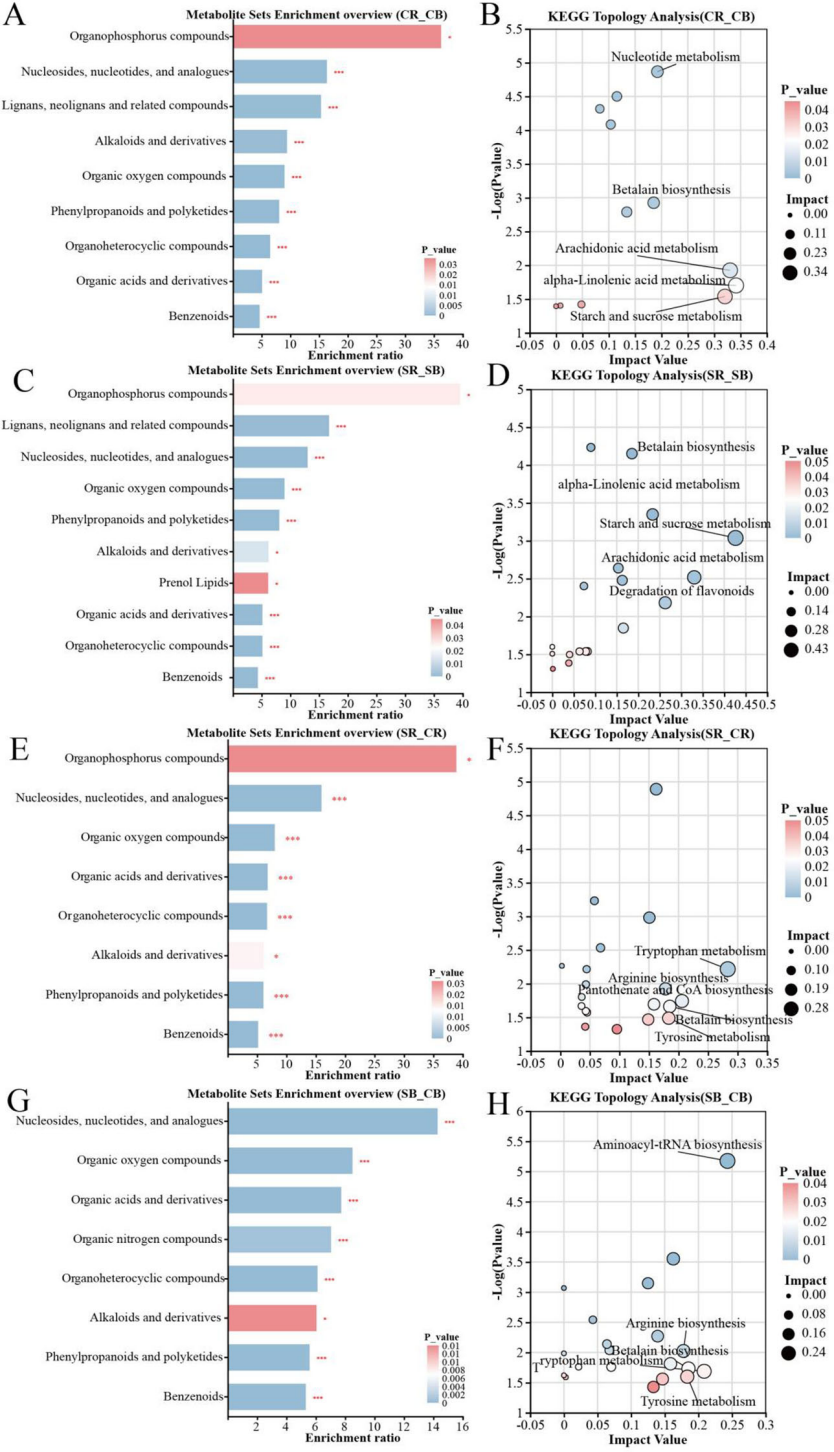
Pathway enrichment (MSEA) and KEGG topology analysis of differential metabolites. **(A, C, E, G)** Overview plots of metabolite set enrichment for CR vs CB, SR vs SB, SR vs CR, and SB vs CB, respectively. **(B, D, F, H)** Bubble plots of KEGG topology analysis for the corresponding pairwise comparisons. CR/CB: Conventional plot rhizosphere/bulk soil; SR/SB: Saline-alkali plot rhizosphere/bulk soil. *P < 0.05, **P < 0.01, ***P < 0.001.

Metabolite set enrichment analysis (MSEA) and KEGG topology analyses were used to elucidate the functional implications of metabolic shifts. Across all comparisons, “Organophosphorus compounds” emerged as the most significantly enriched chemical class. In the CR vs CB comparison (**Figures 9A, B**), this set had the highest enrichment ratio, followed by “Nucleosides, nucleotides, and analogs.” KEGG topology analysis for this group showed that “Nucleotide metabolism” and “Betalain biosynthesis” were the most influential and significantly altered pathways. For the SR vs SB comparison (**Figures 9C, D**), “Organophosphorus compounds” and “Lignans, neolignans and related compounds” were the compound sets with the highest enrichment ratios, while “Betalain biosynthesis” and “alpha-Linolenic acid metabolism” were identified as key enriched pathways. In the crucial SR vs CR comparison (**Figures 9E, F**), “Organophosphorus compounds” and “Nucleosides, nucleotides, and analogs” were again significantly enriched. KEGG topology analysis for this group highlighted “Tryptophan metabolism” and “Arginine biosynthesis” as the most affected pathways (with high impact values and significance), indicating a fundamental shift in microbial protein synthesis potential under saline–alkaline stress. In the bulk soil comparison (SB vs CB, **Figures 9G, H**), “Aminoacyl-tRNA biosynthesis” along with “Arginine biosynthesis” exhibited the highest statistical significance and impact values.

**Figure 10:**
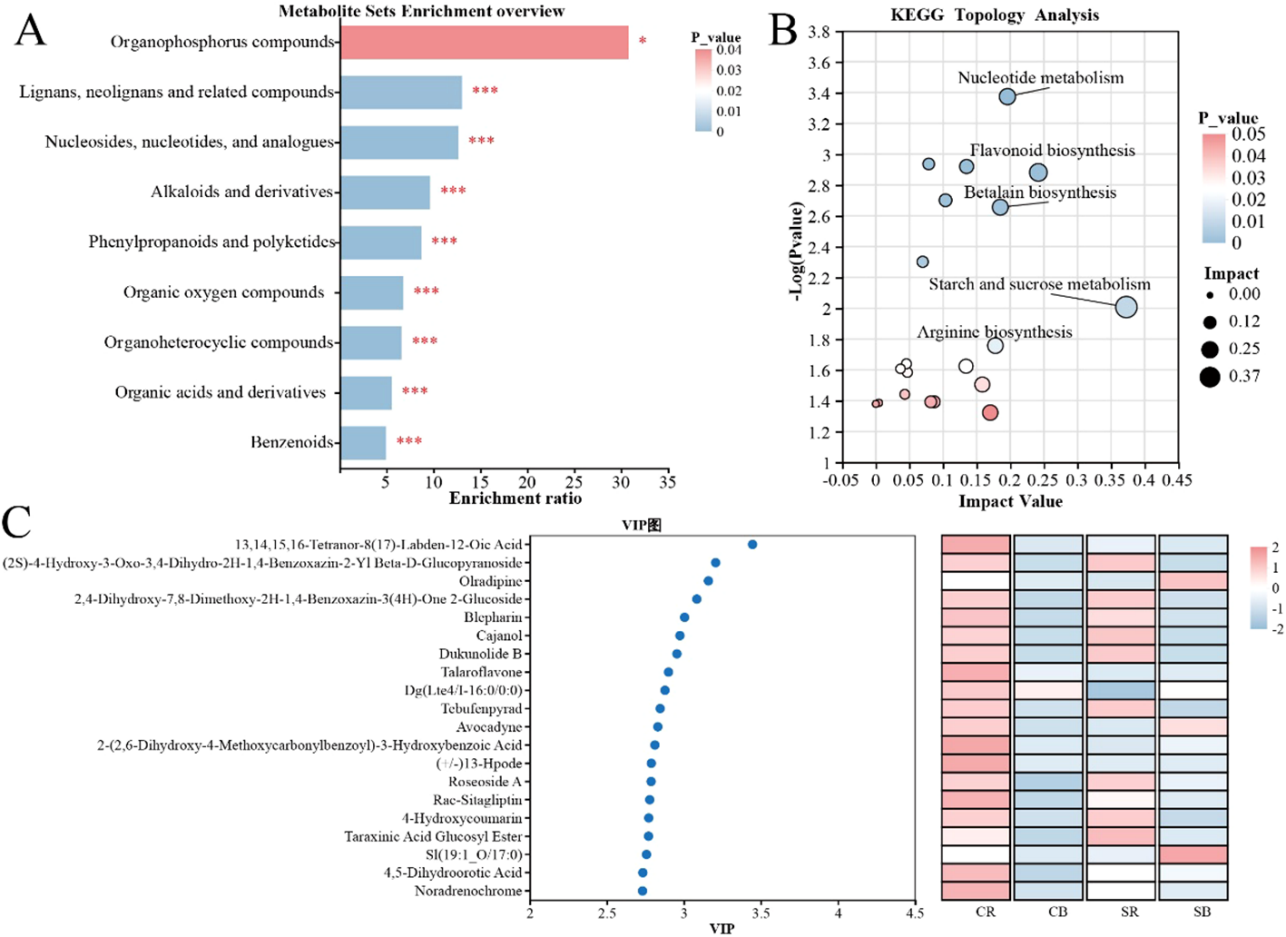
Analysis of differential metabolites and metabolic pathways in rhizosphere soil across treatments. **(A)** Metabolite set enrichment analysis (MSEA) of differential metabolites. **(B)** KEGG pathway enrichment and topology analysis of differential metabolites; bubble size indicates pathway impact value, and color indicates significance level. **(C)** Top 20 differential metabolites with VIP > 2.5 based on OPLS-DA and their relative abundances (Z-score normalized) across different treatments. CR/CB, Conventional plot rhizosphere/bulk soil; SR/SB, Saline-alkali plot rhizosphere/bulk soil. *P < 0.05, **P < 0.01, ***P < 0.001.

We also analyzed the differential metabolites across all four treatments (**Figure 10**). Metabolite set enrichment analysis (MSEA) (**Figure 10A**) showed that “Organophosphorus compounds” exhibited the highest enrichment ratio, a prominent feature of metabolic variation. However, regarding statistical significance, “Lignans, neolignans and related compounds,” “Nucleosides, nucleotides, and analogs,” and “Alkaloids and derivatives” were the most significantly perturbed sets, although their enrichment ratios were lower than that of organophosphorus compounds. Other organic classes, such as “Phenylpropanoids and polyketides” and “Benzenoids,” also showed significant enrichment.

KEGG topology analysis further mapped these metabolites to biological pathways, simultaneously assessing the statistical significance and pathway impact values (**Figure 10B**). “Nucleotide metabolism” was the most significant pathway, which was consistent with the observed enrichment of nucleosides in MSEA. In contrast, although “Starch and sucrose metabolism” had lower statistical significance than that of nucleotide metabolism, it exhibited the highest pathway impact value. Notably, “Flavonoid biosynthesis” and “Betalain biosynthesis” showed both high statistical significance and moderate impact values.

The variable importance in projection (VIP) score plot, combined with a relative abundance heatmap, identified the top 20 key metabolites that influenced the separation among the treatment groups (**Figure 10C**). The metabolite 13, 14, 15, 16-tetranor-8(17)-labden-12-oic acid showed the highest VIP score, followed by (2S)-4-hydroxy-3-oxo-3, 4-dihydro-2H-1,4-benzoxazin-2-Yl β-D-glucopyranoside. The heatmap revealed the unique accumulation patterns of these key metabolites. Top-ranked metabolites (13, 14, 15, 16-tetranor-8(17)-labden-12-oic acid and olradipine) were highly enriched in CR but scarce in saline–alkali samples. Conversely, specific stress-responsive metabolites, such as cajanol, Dukunolide B, tebufenpyrad, and Roseoside A, exhibited specific accumulation in the SR group, highlighting their potential role as biomarkers for rhizosphere salt stress adaptation.

### 3.8 Integrated analysis of soil chemical properties, microbial communities, and rhizosphere metabolites

**Figure 11:**
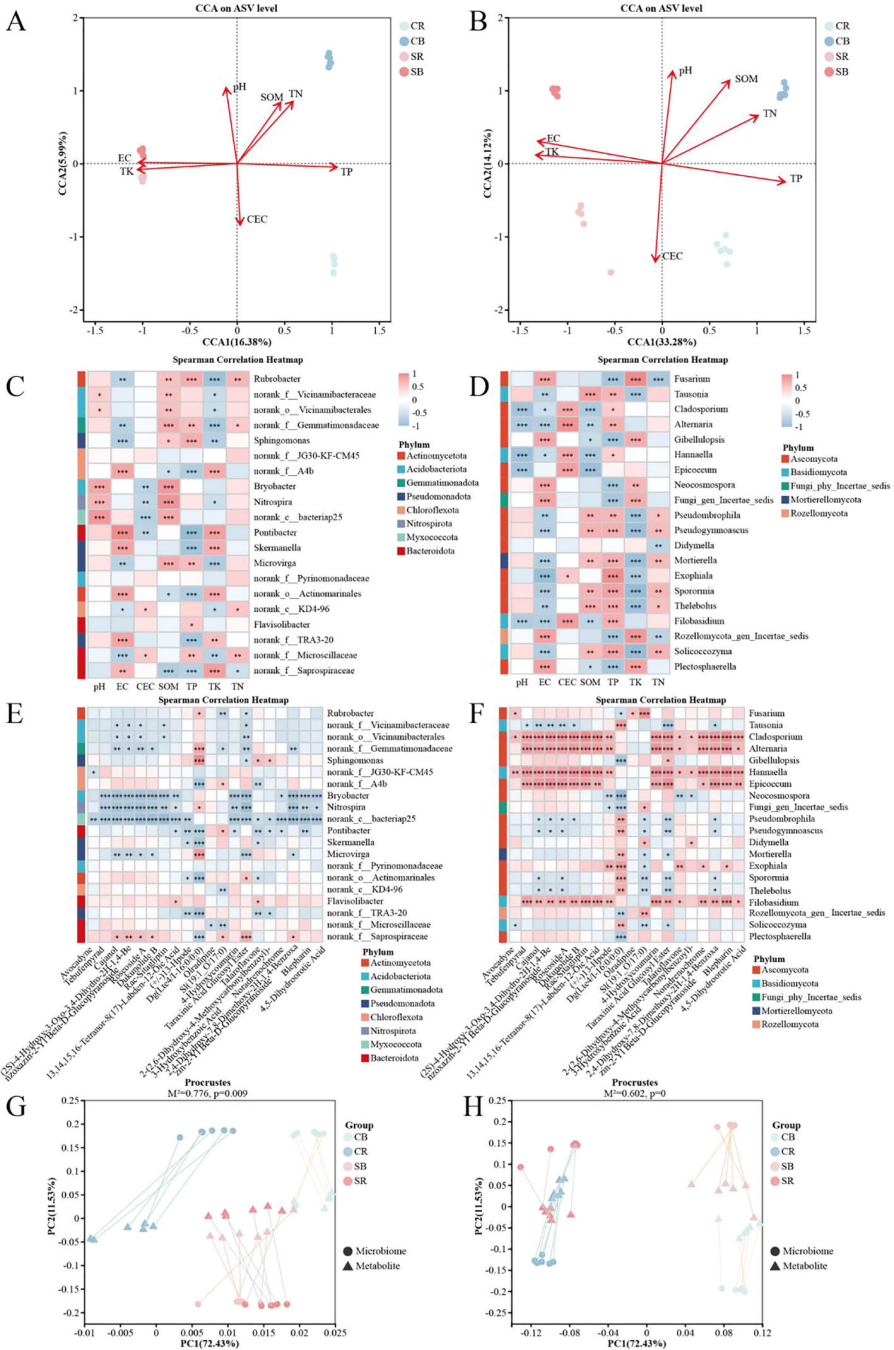
Integrated correlation analysis of soil factors, microbial taxa, and metabolites. **(A, B)** Canonical correspondence analysis (CCA) of bacteria and fungi; **(C, D)** Spearman correlation heatmap between the top 20 dominant bacterial/fungal genera and soil properties; **(E, F)** Correlation heatmap between the top 20 differential metabolites (by VIP) and dominant bacterial/fungal taxa; **(G, H)** Procrustes analysis of the consistency between microbiome and top 20 metabolite (by VIP) profiles. CR/CB, Conventional plot rhizosphere/bulk soil; SR/SB, Saline-alkali plot rhizosphere/bulk soil. *P < 0.05, **P < 0.01, ***P < 0.001.

Canonical correspondence analysis (CCA) indicated that soil chemical properties were the primary drivers of bacterial and fungal community composition (**Figure 11**). For the bacterial community (**Figure 11A**), the first two axes explained 22.37% of the total variation. The samples formed apparent clusters along environmental gradients, with saline–alkali samples (SR and SB) separated from the control samples (CR and CB) along CCA1. The vectors for EC and TK indicated strong positive correlations with saline–alkali samples, whereas SOM, TN, and pH were positively correlated with the control samples. This trend was more pronounced for the fungal community (**Figure 11B**), where the first two axes explained a significantly higher proportion of the variation (47.40%). Driven by opposing vectors for EC, TK, SOM, TN, and TP, an apparent separation was observed between the saline–alkali and control groups. Notably, the CEC vector was orthogonal to the salinity axis, primarily distinguishing the rhizosphere from the bulk soil samples, indicating that the rhizosphere processes maintaining CEC were distinct from the salinity gradient.

Spearman’s correlation heat maps at the genus level revealed the specific ecological preferences of the dominant taxa. An apparent dichotomy was observed in the bacterial communities (**Figure 11C**). Taxa enriched in the control soils, such as *Rubrobacter* and *Nitrospira*, showed significant positive correlations with SOM, TN, and TP, but strong negative correlations with EC and TK. In contrast, the characteristic genera of saline–alkali soils, including *Sphingomonas*, *Pontibacter*, and *norank_f_Vicinamibacteraceae*, exhibited robust positive correlations with EC and TK, but were negatively correlated with SOM. The fungal responses were equally distinct (**Figure 11D**). *Fusarium* showed the strongest positive correlation with EC and TK among all the fungi, indicating its high salt tolerance. Conversely, *Cladosporium*, *Tausonia*, and *Mortierella* exhibited significant positive correlations with SOM and TN, indicating their sensitivity to salt stress and their dependence on high nutrient availability.

Metabolome correlations revealed significant associations between microbial taxa and key differential metabolites (**Figures 11E, F**). Specific metabolites identified as biomarkers in the saline–alkaline rhizosphere (tebufenpyrad, cajanol, Dukunolide B, and taraxinic acid glucosyl ester) were significantly positively correlated with the bacterial genera *Sphingomonas*, *Pontibacter*, and *norank_f_Vicinamibacteraceae* (**Figure 11E**). Fusarium exhibited a strong positive correlation with olradipine in the fungal community (**Figure 11F**). Conversely, *Tausonia* showed a significant negative correlation with taraxinic acid glucosyl esters. *Cladosporium*, *Alternaria*, *Hannaella*, *Epicoccum*, and *Filobasidium* were strongly correlated with metabolites, such as tebufenpyrad, cajanol, Roseoside A, Dukunolide B, and olradipine.

Procrustes analysis was used to test the overall concordance between microbiome and metabolome profiles. Significant correlations were observed between bacterial and fungal communities (**Figures 11G, H**). For bacteria (**Figure 11G**), the sum of the squared Procrustes residuals (*M²*) was 0.776. In contrast, the fit for the fungal community was significantly tighter (**Figure 11H**), with a lower *M²* value (0.602). This suggests that plant roots actively coordinate more synchronous responses between their associated microbiomes and metabolic profiles.

## 4 Discussion

### 4.1 Soil chemical divergence and rhizosphere buffering effect under saline–alkaline stress

Carbonate-type salinization–alkalization, dominated by NaHCO_3_ or Na_2_CO_3_, which is fundamentally different from neutral salt stress, imposes both osmotic stress and nutrient fixation owing to extremely high pH (8.2–10.2) on maize. Our results showed that salinization–alkalization (SR and SB) significantly increased pH (8.36-8.66, moderate carbonate stress) and EC values and led to notable decreases in SOM, TN, and TP content (**Table 1**). This is consistent with recent findings that high alkalinity accelerates organic matter mineralization and inhibits nitrogen and phosphorus pool replenishment in black soil regions (Li et al., 2025a; Xiao et al., 2025). Notably, the SOM content in the saline–alkali rhizosphere was significantly higher than that in the corresponding bulk soil, demonstrating a distinct “rhizosphere buffering effect.” This phenomenon may be attributed to maize roots actively secreting organic acids and protons, neutralizing OH^-^ ions in the high-carbonate environment, thereby creating a relatively favorable environment for nutrient cycling within the rhizosphere microdomain (Shao et al., 2025). The maintenance of CEC in the SR further indicates that root-driven ion exchange processes are crucial for alleviating alkaline toxicity in the immediate root microenvironment (Yang et al., 2025). More importantly, this stable rhizosphere microenvironment provides the core prerequisite for the assembly of stress-resistant microbial communities under stress.

### 4.2 Differential recruitment strategies for bacteria and fungi

High-throughput sequencing results provide a comprehensive understanding of how carbonate-dominated saline–alkaline stress and the rhizosphere collectively influence soil microorganisms and their functional potential. A notable observation in this study was the divergent responses of bacterial and fungal diversity to environmental stresses. Although soil salinization typically leads to decreased microbial richness, the increase in bacterial alpha diversity under saline–alkali treatment, particularly the highest diversity in the SR, suggests a complex recruitment process. This divergent response can be explained by r/K life history strategy: bacteria (mostly r-strategists) have stronger environmental adaptability, while fungi (mostly K-strategists) are more sensitive to high pH stress (Cheng and Wan, 2023). This indicates that plants under abiotic stress actively modulate their root exudates to recruit diverse functional bacteria from the surrounding soil to mitigate stress-induced damage (Zhong et al., 2025). The enrichment of *Pseudomonas* and *Sphingomonas* in the rhizosphere highlighted this directional recruitment. *Sphingomonas* produces extracellular polysaccharides and degrades complex organic compounds, potentially providing a buffering effect for roots against high pH and ionic toxicity (Acuña et al., 2024), and has been shown to promote plant growth in saline-alkaline soils (Li et al., 2024), and its enrichment is further confirmed to be mediated by rhizosphere metabolites in the subsequent correlation analysis.

In contrast, the fungal community diversity substantially declined under saline–alkaline conditions, accompanied by a notable structural shift. The extreme reduction in Sobs and Shannon indices in the SB suggests that carbonate stress presents severe physiological barriers to most soil fungi, consistent with studies showing that saline–alkaline stress significantly reduces microbial community alpha diversity (Tian et al., 2025). However, the diversity in the SR was significantly higher than that in the SB, highlighting the role of the root zone as an important ecological refuge. The dominance of Ascomycota and Basidiomycota in stressed soils indicates community evolution towards groups with higher stress resistance and faster growth rates. Notably, *Fusarium* was identified as the strongest biomarker in SB, and its abundance was significantly higher than that in the control soils, indicating the degradation of soil suppressiveness. *Fusarium* is a notorious soil-borne pathogen. Its proliferation under carbonate stress indicates that salinization–alkalization may increase plant disease risk by altering the competitive balance between saprotrophs and pathotrophs (Zhang et al., 2020). Although the rhizosphere buffering effect alleviated fungal diversity loss, it could not reverse the overall pathogenic transformation trend of the fungal community under stress.

Functional shifts predicted using KEGG and FUNGuild analyses revealed fundamental metabolic trade-offs influenced by the saline–alkaline environments. The increased relative abundance of ABC transporters and two-component systems in the saline–alkali groups indicates that soil bacteria prioritize the reinforcement of survival mechanisms, such as ion homeostasis, nutrient sensing, and signal transduction. This shift occurred at the expense of growth-related processes, as evidenced by a reduction in ribosome biogenesis and protein synthesis pathways. This metabolic restructuring constitutes a key indicator of microbial adaptation to high pH and osmotic stress (Qu et al., 2022). Additionally, FUNGuild elucidated a notable reorganization of fungal trophic modes. The replacement of undefined saprotrophs with complex pathotrophic and parasitic functional guilds in the SB and SR indicated a loss of decomposition function in the soil. The shift towards pathotrophism may represent an adaptive response to nutrient scarcity and alkalinity, wherein fungi adopt endophytic or parasitic lifestyles to acquire resources from the host-protected rhizosphere or stressed plant tissues (Li et al., 2025b). Collectively, these findings suggest that carbonate-type saline–alkaline stress reduces soil health by favoring pathogenic fungi, as well as forces bacterial communities into survival-oriented metabolic shifts, while plants attempt to counteract these negative effects by strategically recruiting protective rhizosphere partners.

The notable reorganization of the microbial community topology further reveals the internal logic of ecosystem coping strategies under saline–alkaline stress. In this study, bacterial networks exhibited increased complexity and connectivity under saline–alkaline pressure, with this intensified structure peaking in the SR, indicating a tendency of bacterial communities to resist environmental disturbances by enhancing interspecies synergy or metabolic coupling (Wang et al., 2024b). The stable positive-to-negative edge ratio in SR bacterial network further confirmed that the enhanced complexity was driven by synergistic interactions between species. The increased average clustering coefficient and average degree suggest that rhizosphere bacteria do not survive in isolation, but construct a synergistic functional alliance with functional redundancy and complementarity. This increase in network complexity is frequently considered a marker of enhanced ecosystem homeostasis, which is beneficial for maintaining key ecological functions such as ion homeostasis (Zhang et al., 2023). In contrast, the fungal network performance showed significant niche differentiation. Although the rhizosphere effect relatively maintained network connectivity, the significant contraction of the fungal network in the SB exposed its topological vulnerability, suggesting that fungi are generally more sensitive to saline–alkaline disturbances than bacteria (Chen et al., 2022). This considerable reduction in the network scale corresponds to the absolute dominance of a few tolerant taxa, such as *Fusarium*, indicating that extreme saline–alkaline environments disrupt the original competitive balance, causing fungal communities to degrade from a diverse, complementary structure to one dominated by limited superior competitors, thereby increasing the potential risk of soil-borne disease outbreaks (Lin et al., 2023). The strengthening of bacterial networks and fragmentation of fungal networks constitute the core characteristics of the soil biotic system under carbonate stress, suggesting that plant roots implement a “cry for help” signaling strategy by secreting specific metabolites and prioritizing the consolidation of a bacterial defense system to compensate for the ecological risks posed by the loss of fungal diversity (Liu et al., 2024).

### 4.3 Impact on metabolites

The apparent separation of PCA and OPLS-DA highlights the considerable influence of carbonate-dominated saline–alkaline stress on the chemical environment of the maize rhizosphere. The first principal component primarily distinguished soil types, whereas the second distinguished niches, indicating that although environmental stress is the main driver, the rhizosphere effect still plays a robust role in maintaining metabolic diversity (Sibalekile et al., 2025). This pronounced separation indicates a sophisticated metabolic remodeling strategy in the maize rhizosphere, where roots are not passively leaking metabolites but actively altering the rhizosphere chemical composition to construct a specific protective niche. This is consistent with the “cry for help” hypothesis, in which plants alter their secretion patterns under stress to recruit beneficial microbes or promote nutrient activation (Parasar et al., 2024).

The most notable finding of the enrichment analysis was the high enrichment of plant-derived organophosphorus compounds. In alkaline soils, inorganic phosphorus is easily fixed by high concentrations of calcium and magnesium ions, leading to severe phosphorus deficiency (Liu et al., 2024). The enrichment of organophosphorus compounds, combined with considerable perturbations in nucleotide metabolism pathways, indicates that maize enhances phosphorus cycling pathways to maintain energy homeostasis. Nucleosides and their analogs are fundamental components for genetic material synthesis, as well as key signaling molecules and phosphorus reservoirs (Keren et al., 2024; Zeng et al., 2025). By accumulating these compounds, maize may recruit specific microorganisms to convert organic phosphorus into available inorganic phosphorus, thereby promoting microbial phosphorus mineralization in the rhizosphere (Wang et al., 2025a).

This study revealed that betalain and flavonoid biosynthesis are the key upregulated pathways in the saline–alkaline rhizosphere. Saline–alkaline stress leads to excessive production of reactive oxygen species (ROS), resulting in lipid peroxidation. The activation of the betalain pathway in maize suggests a surge in nitrogen-containing antioxidant pigments that mitigate oxidative damage (Wang and Sun, 2025). Concurrently, cajanol, an isoflavonoid phytoalexin of plant origin with a high VIP score that specifically accumulates in the rhizosphere, exhibits significant antimicrobial activity and inhibits the growth and proliferation of various pathogenic fungi (Qu et al., 2024). Its enrichment in the rhizosphere may act as a chemical shield, suppressing the expansion of salt-tolerant pathogens, while also serving as a signaling molecule that mediates the recruitment of plant growth-promoting rhizobacteria such as *Sphingomonas*. The substantial shifts in tryptophan metabolism and arginine biosynthesis further indicate the transition of maize metabolism from a growth-oriented mode to a stress-resistant mode. Arginine is a polyamine precursor that can stabilize cell membranes and function as an osmoregulator under salt stress (Nahar et al., 2025). Additionally, amino acid enrichment in the rhizosphere provides essential carbon and nitrogen sources for the microbiome. The specific accumulation of Gly-Asp-Gln in the saline-alkaline rhizosphere (SR) indicates that maize roots may secrete specific peptides to sustain the growth of the recruited salt-tolerant microbial taxa.

### 4.4 Multi-omics integration analysis

Integrated analysis of soil chemical features, microbial community structure, and rhizosphere metabolome provides strong evidence that carbonate-type saline–alkaline stress acts as a primary filter for ecological assembly, while the host plant actively mediates a coordinated survival response through the soil-microbe-metabolite axis (Wang et al., 2025b). CCA evidently showed that EC and TK were the dominant environmental forces driving the differentiation of saline–alkali samples from controls, whereas soil organic matter and nitrogen availability defined the steady state of the non-stressed environment. The explanatory power of environmental vectors for fungal communities was significantly higher than that for bacterial communities, indicating that fungi are more susceptible to direct physiological exclusion caused by high pH and salinity and exhibit more pronounced niche separation. Notably, the orthogonal relationship between CEC and the salinity gradient highlights that the rhizosphere region maintains ion buffering capacity independent of external salt concentration, indicating that root activity acts as a local stabilizer of nutrient bioavailability in harsh chernozem soils (Zhong et al., 2025).

Spearman correlation heat maps further elucidated the specific ecological preferences of the recruited taxa, reinforcing the “cry for help” hypothesis. Robust positive correlations between salt-tolerant genera such as *Sphingomonas* and *Pontibacter* and protective metabolites such as cajanol, Dukunolide B, and tebufenpyrad suggest a directional metabolic recruitment strategy. Cajanol, a specific isoflavonoid phytoalexin, along with other benzenoid compounds, appears to act as both a chemical signal and a defense agent, promoting the colonization of beneficial bacteria while potentially modulating the fungal living environment. Although the direct interaction between cajanol and Sphingomonas awaits experimental validation, numerous studies have demonstrated that plant flavonoids can function as signal molecules to recruit beneficial rhizobacteria, providing indirect support for this proposed mechanism (Bag et al., 2022). This is corroborated by the strong association of these metabolites with salt-sensitive but beneficial fungi, such as *Cladosporium* and *Mortierella*, indicating that plants may attempt to maintain these fungi within the protected rhizosphere niche to sustain nutrient cycling functions (Lei et al., 2025; Shao et al., 2025). Conversely, the dominance of *Fusarium* in saline–alkali soils and its high correlation with TK and specific markers such as olradipine signal the degradation of soil suppressiveness, where pathogenic fungi exploit the stressed physiological state of the host and alkalinity-induced shifts in microbial competition (Zhang et al., 2020).

Procrustes analysis indicated high synchronicity between the biotic and chemical components of the rhizosphere. Fungal communities exhibited tighter coupling than bacterial communities, suggesting that the root-mediated chemical environment and secondary metabolites more directly control fungal assembly. This study finally constructed a complete regulatory framework of “environmental filter-rhizosphere metabolic reprogramming-beneficial bacteria recruitment-stress-resistant network construction” for maize adapting to carbonate saline-alkaline stress. Under carbonate stress, maize roots strategically manage this multi-omics axis to recruit protective bacterial communities and stabilize core fungal taxa, although the inherent proliferation of pathogens, such as Fusarium, remains a notable challenge to long-term soil health. Compared with previous studies, the core innovation of this study is that it systematically reveals the synergistic response mechanism of maize rhizosphere microecosystem to carbonate-type saline-alkaline stress under field *in-situ* conditions, and identifies key signal metabolites mediating beneficial bacteria recruitment. Collectively, these findings suggest that future strategies for managing carbonate-type saline–alkaline land should move beyond traditional chemical remediation towards holistic management of the soil-microbe-metabolite interface, leveraging specific biomarkers, and recruiting functional microorganisms to enhance the intrinsic resilience of agricultural ecosystems (Fang et al., 2025; Qu et al., 2024).

However, this study provides only a single time point snapshot, overlooking seasonal dynamics. Moreover, the observed correlations between microbial taxa and metabolites require experimental validation, and taxonomy-based functional predictions await confirmation via meta-omics approaches. Furthermore, the relationship between Fusarium and crop health parameters was not assessed in the present work and warrants future investigation. Findings from a single site may not be generalizable, necessitating multi-site trials across diverse saline–alkaline regions. Future research should prioritize temporal sampling, causal validation, and the development of metabolite-based biostimulants or microbial consortia for field applications.

## 5 Conclusion

This study indicated that enhancing rhizosphere micro-ecological resilience is a key strategy for addressing carbonate-type saline–alkali soil degradation. Maize roots, through the rhizosphere buffering effect, maintain ion balance and organic carbon stability in the rhizosphere microdomain under extreme pH and osmotic stress. This microenvironment regulation mechanism directionally enriches tolerant bacteria such as *Sphingomonas* and *Pseudomonas* and mitigates the salt-alkali-induced loss of fungal diversity. Microbial co-occurrence network analysis further indicated that saline–alkaline stress enhanced the complexity and synergy of bacterial networks, forming a complementary and functional stress-resistance consortium. Concurrently, rhizosphere metabolic reprogramming activates key pathways, including organophosphorus compound metabolism, flavonoid biosynthesis, and tryptophan and arginine metabolism, and specifically accumulates defensive metabolites such as cajanol, shifting the metabolic pattern from growth-oriented to stress-adaptive. These results systematically revealed the mechanism by which maize roots adapt to saline–alkaline environments by integrating microbiome and metabolome reconstruction, providing a theoretical basis for multi-omics regulation-based ecological remediation and sustainable production of saline–alkaline land. Future research should prioritize temporal dynamics of rhizosphere interactions, experimental validation of key microbe and metabolite linkages, and multi-omics functional assays. Translating these findings into practical strategies, for instance, cajanol may serve as a diagnostic metabolic marker and *Sphingomonas* as a candidate inoculant for rhizosphere management in carbonate-type saline-alkaline soils, will be essential for sustainable saline-alkaline land management.

## Data Availability

The data presented in this study are deposited in two public repositories. Microbial raw sequence data are available in the NCBI SRA database under accession PRJNA1433267, and metabolomics raw data are available in the MetaboLights database under accession MTBLS13956.
